# Multi-Level Representation Learning for Chinese Medical Entity Recognition: Model Development and Validation

**DOI:** 10.2196/17637

**Published:** 2020-05-04

**Authors:** Zhichang Zhang, Lin Zhu, Peilin Yu

**Affiliations:** 1 College of Computer Science and Engineering University of Northwest Normal Lanzhou China

**Keywords:** medical entity recognition, multi-level representation learning, Chinese, natural language processing, electronic medical records, multi-head attention mechanism

## Abstract

**Background:**

Medical entity recognition is a key technology that supports the development of smart medicine. Existing methods on English medical entity recognition have undergone great development, but their progress in the Chinese language has been slow. Because of limitations due to the complexity of the Chinese language and annotated corpora, these methods are based on simple neural networks, which cannot effectively extract the deep semantic representations of electronic medical records (EMRs) and be used on the scarce medical corpora. We thus developed a new Chinese EMR (CEMR) dataset with six types of entities and proposed a multi-level representation learning model based on Bidirectional Encoder Representation from Transformers (BERT) for Chinese medical entity recognition.

**Objective:**

This study aimed to improve the performance of the language model by having it learn multi-level representation and recognize Chinese medical entities.

**Methods:**

In this paper, the pretraining language representation model was investigated; utilizing information not only from the final layer but from intermediate layers was found to affect the performance of the Chinese medical entity recognition task. Therefore, we proposed a multi-level representation learning model for entity recognition in Chinese EMRs. Specifically, we first used the BERT language model to extract semantic representations. Then, the multi-head attention mechanism was leveraged to automatically extract deeper semantic information from each layer. Finally, semantic representations from multi-level representation extraction were utilized as the final semantic context embedding for each token and we used softmax to predict the entity tags.

**Results:**

The best F1 score reached by the experiment was 82.11% when using the CEMR dataset, and the F1 score when using the CCKS (China Conference on Knowledge Graph and Semantic Computing) 2018 benchmark dataset further increased to 83.18%. Various comparative experiments showed that our proposed method outperforms methods from previous work and performs as a new state-of-the-art method.

**Conclusions:**

The multi-level representation learning model is proposed as a method to perform the Chinese EMRs entity recognition task. Experiments on two clinical datasets demonstrate the usefulness of using the multi-head attention mechanism to extract multi-level representation as part of the language model.

## Introduction

### Background

**Figure 1:**
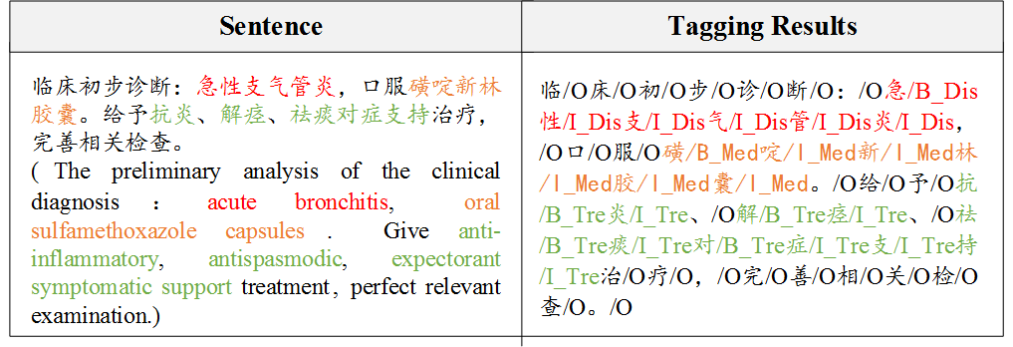
A tagging example of Chinese electronic medical records.

Electronic medical records (EMRs) comprise patients’ health information. Diagnostic accuracy can be improved by making full use of the available information in EMRs. Medical entity recognition (ER) is a fundamental task of medical natural language processing (NLP) and is usually treated as a sequence labeling problem [1]. As shown in Figure 1, in which three predefined entity categories are disease, drug, and treatment, when using the BIO (beginning of the noun phrase, middle of the noun phrase, and not a noun phrase) labeling mode to tag Chinese EMRs, the candidate label set contains seven types: B-Dis (disease), I-Dis, B-Med (medicine), I-Med, B-Tre (treatment), I-Tre, and O.

Generally, the methods of ER can be divided into two categories. The first category leverages rules and dictionaries to represent linguistic features and domain knowledge to identify clinical entities [2]. The second category is based on traditional machine learning and neural networks [3-8]; this type of method greatly improves the performance of ER models but requires large-scale labeled data during model parameter training. In the medical field, creating annotation datasets is restricted by professional knowledge and legal regulations, so the lack of annotated corpora becomes one of the greatest technical challenges. At present, ER attracts a lot of attention from the field to improve the representation learning capability of current methods. Research studies have demonstrated that using embedding techniques can help solve the problem of missing supervised data in NLP tasks, including the factorization methods of Global Vectors (GloVe) [9], the neural methods of word2vec [10] and fastText [11], and more recent dynamic methods that take into account the context, such as Embeddings from Language Models (ELMo) [12] and OpenAI Generative Pre-trained Transformer (GPT) [13]. Those embedding technologies can capture the context of semantics in unsupervised data and generate different vector representations of the same word in different contextual situations.

Among them, Bidirectional Encoder Representations from Transformers (BERT) [14] integrates many top ideas of language models and gives a particularly prominent performance. Transform-block is a feature extractor and learns different types of abstract granularity information. Multi-layer information is iterated layer by layer to generate embedding representation. In the actual training process, most downstream tasks take BERT's last embedding vector as the input of the model. However, studies found that different NLP tasks have different characteristics of requirements. Therefore, combining task features into the language model can reduce the loss of extracted information by the feature extractor and improve the utilization of language models. For example, Peters et al [12] explicitly showed that the lower layer fits into the local semantic relationships, the higher layer is suitable for longer-range relationships, and the final layer specializes in the language model. Peters et al [15] also showed that combining all semantic internal states models, by using a weighted-sum method to represent the vector of a word, can enrich the characteristics of the word in learning deep contextualized embedding representations. Because the Chinese ER task focuses on word granularity information, this is a straightforward way to use the information extracted from the low-layer representation.

In this work, we tackle representation using the BERT language model. Our objective is to extract each layer of semantic information using feature extractors. We constructed a multi-level representation learning model for the optimal integration of information. Our contributions can be summarized as follows:

1. We manually annotated a new Chinese EMR (CEMR) corpus for ER tasks. Moreover, we propose a multi-level representation learning model to mine hidden representation.
2. The proposed model takes advantage of the multi-head attention mechanism to integrate more suitable information from each layer and can perform as a state-of-the-art method on two clinical text datasets.
3. The best F1 score achieved by the experiment was 82.11% on the CEMR corpus and significant improvement on the CCKS (China Conference on Knowledge Graph and Semantic Computing) 2018 benchmark dataset was attained.

### Chinese Electronic Medical Record Dataset: A Newly Constructed Corpus

Large labeled datasets are not always readily accessible. To facilitate the research on the ER task of the Chinese EMRs and future work in related topics, we constructed a new manually annotated CEMR dataset. The normalization of the labeling process refers to a large number of annotation guidelines [16]. All EMRs came from Third-Class A-Level hospitals in Gansu Province, China, which contained 80,000 EMRs across 14 departments. Manual labeling of 4000 medical records provided the data for ER experiments. Table 1 shows the data distribution of the 14 hospital departments. The CEMR corpus contains six types of entities: disease (Dis), symptom (Sym), test, treatment (Tre), medicine (Med), and abnormal inspection result (Abn). The categories are defined as follows:

1. Disease: refers to a specific abnormal pathological condition. This abnormal life condition is caused by disorders of self-regulation, such as diabetes.
2. Symptom: refers to subjective feelings described by patients or objective facts observed externally, such as abdominal distension.
3. Test: includes examination procedures, items, and equipment to collect and confirm more information about the disease or symptom, such as electrocardiogram.
4. Treatment: refers to a treatment program or intervention to treat diseases or relieve symptoms, such as neurotrophic treatment.
5. Medicine: refers to a chemical substance used to prevent and treat diseases or to strengthen the body and improve mental state, such as insulin.
6. Abnormal inspection result: refers to an abnormal change or inspection result observed by doctors or by examination equipment, such as a little sputum sound.

Before labeling the data, private information was removed in the EMRs, such as patients’ names, addresses, and hospital IDs. In the process of labeling samples, the annotation tool is developed specifically for the ER task. Moreover, some strategies have been developed to create high-quality annotated data. For example, the annotation samples will be randomly checked at any time.

**Table 1:** Electronic medical record (EMR) data distribution by department.

Department	EMR count, n (%)
Neurosurgery	77 (1.93)
Neurology	77 (1.93)
Cardiology	77 (1.93)
Gynecology and obstetrics	77 (1.93)
Andrology	77 (1.93)
Respiratory medicine	77 (1.93)
Cardiovasology	77 (1.93)
Hepatobiliary surgery	77 (1.93)
Ophthalmology	77 (1.93)
Orthopedics	77 (1.93)
Gynecology	101 (2.53)
Pediatrics	232 (5.80)
Internal medicine	970 (24.25)
Surgery	1495 (37.38)
Other	432 (10.80)
Total	4000 (100)

## Methods

### Overview

The goal of the ER task is to provide the model with an EMR and its semantic types, so that it can extract and classify all characters in the text. The proposed model consists of three stacked layers: the input layer, the feature extraction layer, and the output layer.

**Figure 2:**
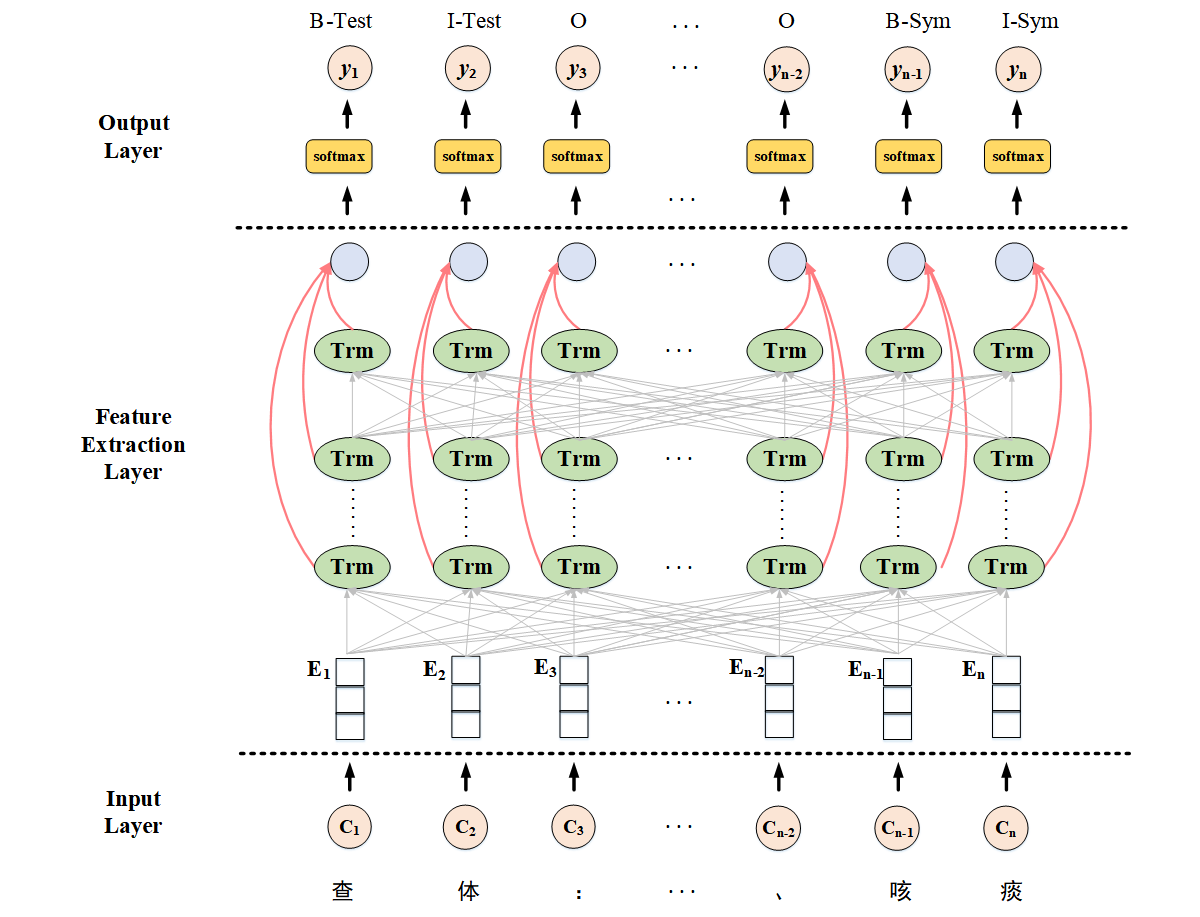
Multi-level representation learning for ER model. B-Sym: beginning of the noun phrase for the symptom entity; B-Test: beginning of the noun phrase for the test entity; C: input sentence; E: input embedding; I-Sym: middle of the noun phrase for the symptom entity; I-Test: middle of the noun phrase for the test entity; O: not a noun phrase; Trm: transform-block; y: output sentence’s predicted tag sequence.

As shown in Figure 2, the model first used the BERT language model to extract the semantic representations. Then, the multi-head attention mechanism was leveraged to automatically extract deeper semantic information from each layer. Finally, the semantic information from the multi-level representation extraction was utilized as the final semantic context embedding for each token and was input into the softmax layer to predict the entity tag. The input sentence was denoted as C = (c_1_, c_2_, c_3_, ..., c_n_), where c_n_ represented the *n*-th character in sentence C of the Chinese EMR. Correspondingly, the output sentence’s predicted tag sequence was denoted as Y = (y_1_, y_2_, y_3_ ..., y_n_), where y_n_ belonged to one of the sets: B-Dis, I-Dis, B-Sym, I-Sym, B-Test, I-Test, B-Tre, I-Tre, B-Med, I-Med, B-Abn, I-Abn, or O. In the following text, we introduce the BERT language model and describe the proposed multi-level representation learning model.

### Bidirectional Encoder Representations From Transformers

BERT was designed to learn deep bidirectional representations by jointly conditioning both the left and right contexts in all layers. It was based on multi-layer bidirectional encoder transformers and could be used for different architectures. When given a character-level sequence C = (c_1_, c_2_, c_3_, ..., c_n_), BERT was formulated as follows:

*h_1_* = *E_Token_* + *E_Segment_* + *E_Position_* (1)

*h_l_* = *Trm*(*h_l–1_*) (2)

*Y^BERT^* = *Softmax*(*w_O_h_L_* + *b_O_*) (3)

where *h*_1_ represents input embedding for a sequence and is made up of *E_Token_*, *E_Segment_*, and *E_Position_*, which mean token, segment, and position for a sentence, respectively. The BERT leverage transformer is the feature extractor. *Trm* is a transform-block that includes self-attention, the fully connected layers, and the output layer. The current *l* layer hidden state came from the upper *l–1* layer and *L* was the last layer. *Y^BERT^* denotes the output layer that predicts the sequence labels. In the above equations, *w_O_* denotes the function weight and *b_O_* is the function bias. All parameters of the transform-block were trained in advance on a large document-level corpus using a masked language model and were fine-tuned by predicting task-specific labels with the output layer to maximize the log-probability of the correct label.

### Multi-Level Representation Learning for Entity Recognition

**Figure 3:**
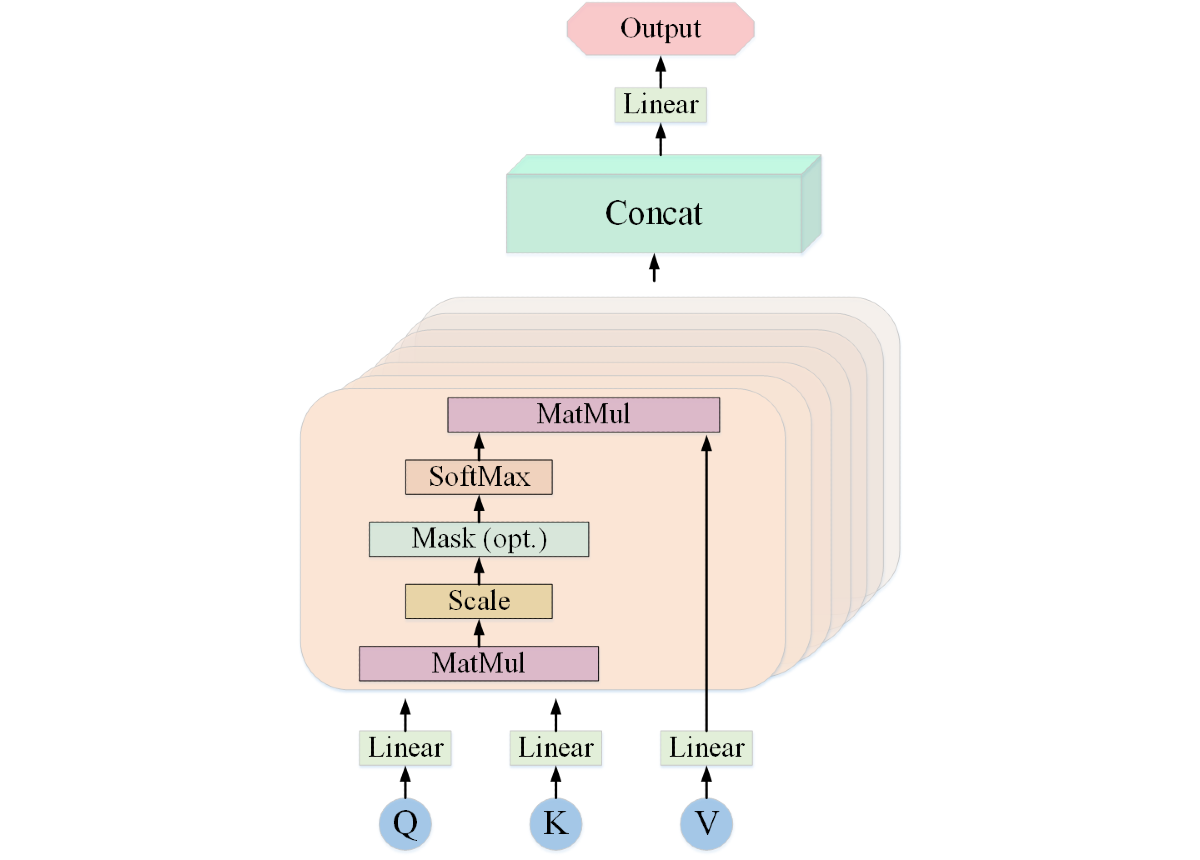
Multi-head attention mechanism. K: key; Q: query; V: value.

The Multi-Level Representation Learning for ER model (Multi-Level ER) could automatically integrate deeper semantic information from all layers of the feature extractor for ER task. The proposed language model took advantage of the multi-head attention mechanism. Multi-head attention is a special type of attention that allowed the model to focus on different positions of subspace representation information and could learn more about the connections between internal elements. Figure 3 shows the calculation process of the multi-head attention mechanism when calculating the weight of the transform-block output knowledge. The query (Q), key (K), and value (V) in the transform-block were calculated. The process of acquiring Q, K, and V could be written as follows:

*H* = *Concat*(*h_1_*, *h_2_*, *h_3_*, ..., *h_L_*) (4)

*Q* = *w_Q_h_L_* + *b_L_* (5)

*K* = *w_K_H* + *b_K_* (6)

*V* = *w_V_H* + *b_V_* (7)

where *h_L_* denotes the hidden state of the final layer of the transform-block. The parameters *w_Q_*, *w_K_*, and *w_V_* are weight matrices. The parameters *b_L_*, *b_K_*, and *b_V_* are bias matrices. The attention function is calculated as follows:

*head_i_* = *Softmax*(*Q_L_K^T^*/*√d*)*V* (8)

where *head_i_* means the *i*-th head. *Q_L_* is the query key value of the last *L* layer. *√d* is used to control the order of magnitude of calculation results and *d* donates the dimension of the *K* vector. In this work, we used multi-head attention, as introduced in the following equation:

*E* = *Concat*(*head_1_*, *head_2_*, *head_3_*, ..., *head_l_*)*w_h_* + *b_h_* (9)

where *w_h_* is used to balance the head weight. For the final layer of the network, we pass the results into a fully connected layer with a softmax function, as follows:

*Y^Multi-Level ER^* = *Softmax*(*w_O_E* + *b_O_*) (10)

where *w_O_* is the output weight matrix and *b_O_* is the bias of the output layer.

### Experiment

This model was supported by multiple sets of comparative experiments. Each group of experiments was repeated three times, and the result in the middle of the ranking was taken as the final result.

#### Dataset and Evaluation Criteria

We evaluated the proposed model on two datasets: the CCKS 2018 dataset and the CEMR dataset. The CCKS 2018 dataset was adopted from the Chinese EMR named ER task at the CCKS, which included 1000 admission records. In the experiment, 600 records were used as training data and the remaining were test data. Comparative experiments were made on the new CEMR corpus and contained 4000 documents. We further split the corpus set by 60%, 20%, and 20% as training, validation, and test sets, respectively. Table 2 shows the distribution of documents in two datasets.

**Table 2:** Components of the two datasets.

Dataset	Number of records per set			
	Total	Training set	Validation set	Test set
CEMR^a^ dataset	4000	2400	800	800
CCKS^b^ 2018	1000	600	N/A^c^	400

^a^CEMR: Chinese electronic medical record.

^b^CCKS: China Conference on Knowledge Graph and Semantic Computing.

^c^Not applicable; because the comparison method does not divide the validation set on the CCKS dataset, we have kept this the same as the original experiment to make the comparison fair.

To evaluate the performance of all prediction methods fairly, the results were validated by precision (P), recall (R), and F1 scores (F1) as measurements to evaluate the recognition effectiveness of the model; these were defined as follows:

P = TP/(TP + TF) (11)

R = TP/(TP + FN) (12)

F1 = (2 × T × P)/(P + R) (13)

An entity is annotated as correct when its category and boundary are fully labeled correctly. TP is the count of entity labels presenting the same labels as gold standard labels, FP is the count of recognized entities marked incorrectly in the results, and FN is the count of the gold standard entities that are not present in the results of the indicator.

#### Parameter Setup

Hyperparameter configuration was adjusted according to the performance on the described validation sets. We used a publicly available pretraining language representation model, namely the BERT_BASE-Chinese-uncased_. This model has 12 layers, 768 hidden layers, and 12 heads. The multi-head attention mechanism was utilized to automatically integrate all layers of information. By comparing experimental results with different head numbers, we had set the head number to 12. We fine-tuned the model over 10 epochs with a batch size of 32. The maximum training sentence length was 64. The model was trained with the AdamW optimizer with a learning rate of le-5 and we applied a dropout rate of 0.3.

## Results

### Overview

We summarized the overall performance by computing the F1 score; the results are illustrated in Table 3. On the CEMR dataset, we compared the multi-level ER learning model with previous classic methods, including conditional random field (CRF), convolutional neural network (CNN)+bidirectional long short-term memory (BiLSTM)+CRF, lattice long short-term memory (LSTM), and BERT. We found that the proposed model is better than state-of-the-art baseline methods, with F1 scores of 0.94% to 4.9%. Our multi-level ER learning model had improved by 1.48% in its *P* value, 0.47% in its R value, and 0.94% in its F1 score compared to the BERT model. The result also demonstrated that pretraining the multi-level ER learning language model was highly effective for task-specific Chinese EMR ER.

**Table 3:** Comparison of method performance on the Chinese electronic medical record (CEMR) dataset.

Method	*P* value (%)	R value (%)	F1 score (%)
Conditional random field (CRF)	88.57	68.43	77.21
CNN^a^+BiLSTM^b^+CRF	81.51	76.92	79.15
Lattice long short-term memory (LSTM)	88.60	74.48	80.93
Bidirectional Encoder Representations from Transformers (BERT)	83.73	78.76	81.17
Multi-level representation learning for entity recognition (multi-level ER)	85.21	79.23	82.11

^a^CNN: convolutional neural network.

^b^BiLSTM: bidirectional long short-term memory.

We also applied our model to the widely used benchmark CCKS 2018 dataset and used the same data split to compare it. Huang et al [17] proposed a BiLSTM-CRF model for sequence tagging and Cai et al [18] was based on the self-matching attention mechanism (SM) and proposed an SM-LSTM-CRF model design for the named ER task. The results are shown in Table 4. Under the condition of not needing any external resources, the proposed multi-level ER learning model already outperformed the previous SM-LSTM-CRF model by 3.1% on the F1 score.

**Table 4:** Comparison of method performance on the China Conference on Knowledge Graph and Semantic Computing 2018 dataset.

Method	*P* value (%)	R value (%)	F1 score (%)
BiLSTM^a^-CRF^b^ [17]	65.68	69.04	67.32
SM^c^-LSTM-CRF [18]	80.54	79.61	80.08
Multi-level representation learning for entity recognition (multi-level ER)	83.90	82.47	83.18

^a^BiLSTM: bidirectional long short-term memory.

^b^CRF: conditional random field.

^c^SM: self-matching attention mechanism.

### The Effect of Assembling Methods

We compared the effects of different assembling methods on model performance to verify the ability of the multi-head attention mechanism to combine hierarchical information. As listed in Table 5, we first applied concatenation that directed the horizontal concatenated tensors; the F1 score was 81.51%. We then adopted the sum average method to get an F1 score of 81.11%. We finally adopted the multi-head attention method, given that it had the best overall performance compared to several other methods we evaluated. The results showed that integrated hidden information can acquire more suitable representation; the multi-head attention mechanism can be leveraged to automatically extract deeper semantic information from each layer, which is the most effective assembling method.

**Table 5:** The effect of assembling methods.

Assembling method	*P* value (%)	R value (%)	F1 score (%)
Concatenation	84.22	78.97	81.51
Sum average	83.27	79.06	81.11
Multi-head attention mechanism	85.21	79.23	82.11

### The Effect of Extraction Layer Numbers

To examine the impact of extraction layer numbers on model performance, we performed comparative experiments using various extraction layer numbers; the results are shown in Table 6. It was observed that the performance of all layers was superior to that of the other numbers of layers, which introduced multi-level ER into the language model and enhanced model performance. By and large, the tendency was that performance improved as the number of extracting layers increased. However, we also discovered that extracting the last four layers gave higher F1 scores than extracting the last six or two layers. The analysis showed that the results were closely related to the specific dataset. Of course, as the number of layers increased, parameters required by the neural network also increased significantly. Therefore, when there was a high demand for speed on the model, we could select a structure that included the last four layers to optimize time efficiency.

**Table 6:** The effect of extracted layer numbers.

Extraction layer number	*P* value (%)	R value (%)	F1 score (%)
Total layers	85.21	79.23	82.11
The last six layers	85.15	78.65	81.77
The last four layers	85.50	78.68	81.95
The last two layers	84.51	78.68	81.49

### The Effect of Dataset Size

**Figure 4:**
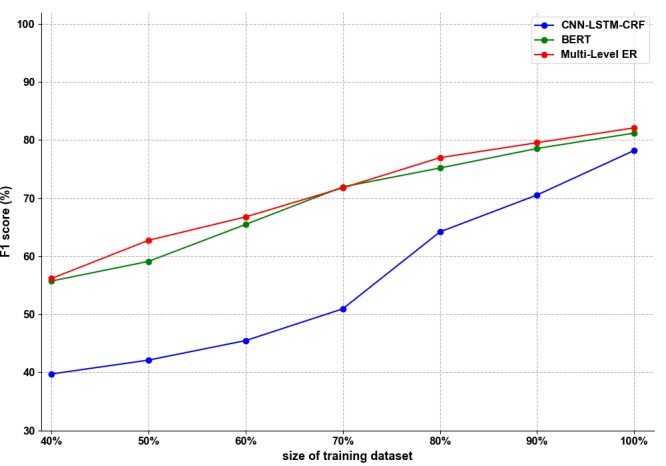
The effect of dataset size. BERT: Bidirectional Encoder Representations from Transformers; CNN: convolutional neural network; CRF: conditional random field; LSTM: long short-term memory; Multi-Level ER: multi-level representation learning for entity recognition.

Figure 4 shows the impact of the dataset size on model performance. Horizontal coordinates represent the size of the training dataset and vertical coordinates indicate the F1 scores. During the experiment, we used different sized corpora to train the CNN-LSTM-CRF, BERT, and multi-level ER models. The figure shows that as the training dataset increased, the performance of the models also improved. In reality, we had a limited number of datasets, and models were unlikely to reach saturation. Therefore, the impact of dataset size on performance was particularly critical. We found that the CNN-LSTM-CRF model performance was sharply affected by the size of the dataset when the training set increased from 70% to 100%. Inversely, the BERT model and the multi-level ER model were less influenced by the training dataset size, and our proposed multi-level RE model outperformed the BERT model.

## Discussion

### Case Studies

**Figure 5:**
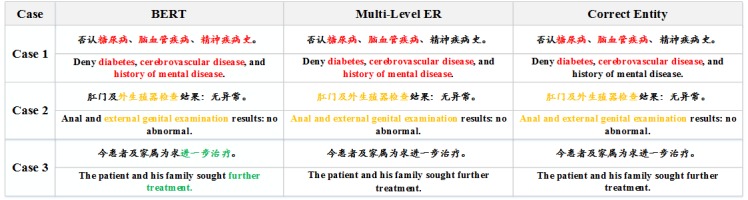
Case studies comparing the multi-level representation learning for entity recognition (Multi-Level ER) model with the Bidirectional Encoder Representations from Transformers (BERT) model.

To show that our model was able to solve the challenge of integrating representation information, three case studies comparing the multi-level ER model with the BERT model are shown in Figure 5. Several obvious trends emerged from the comparative experiments. Most generally, when the word “disease” is included within the medical history, it is mistaken for a disease. For example, case study 1 in Figure 5 shows that “history of mental disease” is recognized as a disease. Case study 2 in Figure 5 shows that when “anal” and “external genitals” appear together before the examination, the system will only identify the adjacent area to be tested. The descriptions with the obvious word “treatment” are identified as a treatment in case study 3 of Figure 5.

We found that the BERT model’s embedding technology improves the performance of the ER model in Chinese EMRs; however, using information from only the last layer of the feature extractor in the language model did not achieve the best experimental results. Our proposed multi-level ER model combines the information from each layer of the feature extractor and selects the most suitable, long-term, syntactic, relationship information for the ER task, which greatly improves the performance of the model.

### Related Work

ER tasks attract a large amount of scholastic attention. The development of deep learning methods has resulted in a breakthrough regarding these tasks. CNN and recurrent neural network (RNN) models have emerged one after another; the attention mechanism and transfer learning were applied to the model. Wu et al [19] utilized a CNN model to generate features represented by several global hidden nodes. Both local features and global features were then fed into a standard affine network to recognize named entities in clinical text. Ju et al [20] used an LSTM neural model to identify nested entities by dynamically stacking flat, named ER layers. Rei et al [21] applied the attention mechanism to dynamically decide how much information to use from a character-level or word-level component in an end-to-end model. Lee et al [22] applied transfer learning in named ER by training a model on source task and using the trained model on the target task for fine-tuning. Peng et al [23] proposed a method where the prediction model was based on BiLSTM, which was taken as the source task of transfer learning. For the ER task in clinical notes, Bharadwaj et al’s [24] work centered on effectively adapting these neural architectures toward low-resource settings using parameter transfer methods.

Language models can capture the syntactic and semantic information of words from a large number of unlabeled texts, which alleviates the problem of an insufficiently annotated corpus in special domains. Peters et al [12] used a language model to obtain a deep contextualized word pretraining representation called ELMo and improved the accuracy of six NLP tasks. Radford et al [13] proposed the GPT for language understanding tasks. For text classification and sequence labeling tasks, the transfer ability is better. Devlin et al [14] proposed the pretraining of deep bidirectional transformers for language understanding (ie, BERT); it captured true directional context information, sweeping 11 NLP tasks through pretraining and fine-tuning.

Our motivation is to seize the optimal information from each layer of a feature extractor to suit a given task. Takase et al [25] employed intermediate layer representation, including input embedding, to calculate the probability distributions to solve a ranking problem in language generation tasks. Kaneko et al [26] demonstrated that learning suitable representation came from different layers in grammatical error detection tasks. Therefore, we tracked their work and found the issue in the ER task in Chinese EMRs.

### Conclusions

We propose a novel, multi-level, representation learning model for ER of Chinese EMRs-the multi-level ER model. We compared our model with state-of-the-art models and observed comparable performance without any external syntactic tools. The results showed that the use of the multi-head attention mechanism can effectively integrate deep semantic information from each layer of the feature extractor. In the future, we plan to apply multi-level ER to other language representation models in order to obtain even greater improvement.

## Abbreviations

Abn: abnormal inspection result
BERT: Bidirectional Encoder Representations from Transformers
BiLSTM: bidirectional long short-term memory
BIO: beginning of the noun phrase, middle of the noun phrase, and not a noun phrase
CCKS: China Conference on Knowledge Graph and Semantic Computing
CEMR: Chinese electronic medical record
CNN: convolutional neural network
CRF: conditional random field
Dis: disease
ELMo: Embeddings from Language Models
EMR: electronic medical record
ER: entity recognition
F1: F1 score
GloVe: Global Vectors
GPT: Generative Pretraining Transformer
K: key
LSTM: long short-term memory
Med: medicine
multi-level ER: multi-level representation learning for entity recognition
NLP: natural language processing
P: precision
Q: query
R: recall
RNN: recurrent neural network
SM: self-matching attention mechanism
Sym: symptom
Tre: treatment
V: value
